# Membrane flex loop-assisted peeling and giant flap creation for primary repair of idiopathic macular holes: a pilot study

**DOI:** 10.1186/s40942-025-00767-1

**Published:** 2025-12-01

**Authors:** Rodrigo Jorge, Victor Bellanda, Arthur S. Zupelli, Moises Moura de Lucena, Letícia O. Audi, Ingrid U. Scott, Antonio Marcelo Barbante Casella

**Affiliations:** 1 Ophthalmology Division, Ribeirão Preto Medical School, University of São Paulo, 3900 Bandeirantes Ave., Ribeirão Preto, SP 14049 – 900 Brazil; 2 Cole Eye Institute, Cleveland Clinic, Cleveland, OH USA; 3 Departments of Ophthalmology and Public Health Sciences, Penn State College of Medicine, Hershey, PA USA; 4 Department of Surgery, Londrina State University, Londrina, PR Brazil

**Keywords:** Macular hole, Internal limiting membrane, Membrane loop, Vitrectomy, Retinal surgery

## Abstract

**Background:**

Idiopathic macular holes are commonly treated with pars plana vitrectomy and internal limiting membrane (ILM) peeling, which achieves high closure rates in smaller holes but is less effective for large or chronic cases. Alternative techniques, such as inverted or free flaps, may improve outcomes but often involve forceps manipulation, which can damage retinal tissue. The membrane loop device (FINESSE^®^ Flex Loop; Alcon), designed for atraumatic manipulation of the ILM, has not been evaluated for giant flap creation. This study reports the anatomical and functional outcomes of macular hole repair using a giant ILM flap created exclusively with the membrane loop.

**Methods:**

This prospective, single-arm interventional case series included patients with large (minimal linear diameter > 400 μm) or chronic (≥ 6 months) idiopathic macular holes. All underwent standard 25-gauge vitrectomy with creation of a 2–3 mm ILM flap using the membrane loop without forceps. The primary endpoint was anatomical closure at day 14. Secondary outcomes included change in best-corrected visual acuity (ETDRS letters), proportion of eyes gaining ≥ 15 letters, and need for reoperation over six months.

**Results:**

Eight eyes from eight patients (median age, 69 years; range, 45–77) were enrolled. Baseline median minimal linear diameter was 400 μm, and median symptom duration was 11 months. Six of eight holes (75%) were closed after primary surgery, although one recurred after two months. The two eyes that were refractory and the one that recurred subsequently achieved closure after reoperation, resulting in a final closure rate of 100% at six months. Median visual acuity improved from 20 to 55 letters, corresponding to a gain of 33 letters (95% CI, + 5 to + 48; *P* = 0.018). Six eyes (75%) gained at least 15 letters, and no intraoperative or postoperative complications occurred.

**Conclusions:**

Membrane loop-assisted giant ILM flap creation yielded a 75% primary and 100% final macular hole closure rate after reoperation, with consistent visual improvement in this pilot series of patients with large or chronic idiopathic macular holes. By minimizing retinal trauma and eliminating the need for forceps, this technique may represent a safe and effective surgical alternative. Larger, comparative studies are warranted to confirm these preliminary results, elucidate long-term anatomical and functional outcomes, and objectively quantify potential differences in retinal trauma relative to traditional forceps-assisted flap techniques.

**Supplementary Information:**

The online version contains supplementary material available at 10.1186/s40942-025-00767-1.

## Background

Idiopathic macular holes (IMH) are primarily managed with pars plana vitrectomy (PPV) and internal limiting membrane (ILM) peeling to relieve vitreoretinal traction and promote anatomical closure. This approach achieves high closure rates in eyes with small to medium-sized holes; however, complications such as retinal nerve fiber layer damage and associated ganglion cell and inner plexiform layer changes may impact visual outcomes [1–5]. 

Recent advances in surgical techniques have sought to overcome the limitations of conventional ILM peeling, particularly for large or chronic macular holes, where closure rates remain suboptimal [6–8]. The use of inverted or free ILM flaps has emerged as a promising strategy to improve anatomical closure and visual outcomes. Studies suggest these flaps act as scaffolds for cellular proliferation and tissue repair, thereby enhancing the likelihood of successful closure compared with traditional methods [6, 7]. 

Despite showing promising results, ILM flap procedures may induce retinal alterations, particularly in the outer retina, and delay the recovery of structures such as the external limiting membrane and ellipsoid zone [9]. Such changes are often linked to instrument-tissue interactions, especially with direct grasping methods like the “pinch-and-peel” technique or the use of diamond-dusted scrapers [10]. The membrane loop (FINESSE^®^ Flex Loop; Alcon, Fort Worth, TX, USA) is specifically designed for ILM manipulation but has not been systematically evaluated as a primary tool for creating giant retinal flaps in IMH surgery. This study addresses that gap by prospectively evaluating the feasibility and short-term safety of primary IMH repair using the giant retinal flap technique performed exclusively with the membrane loop.

## Methods

**Fig. 1:**
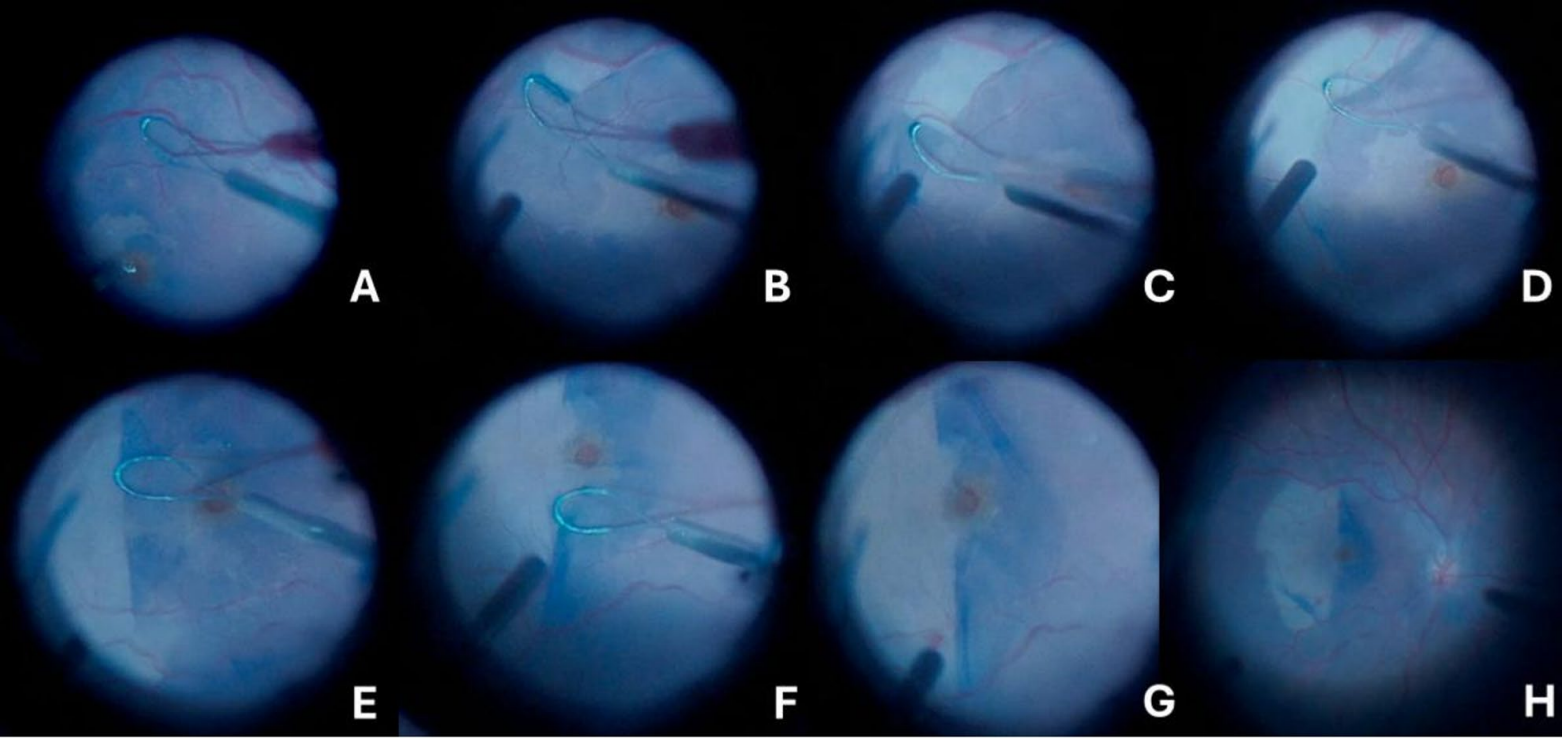
Intraoperative steps of giant internal limiting membrane (ILM) flap creation using the membrane loop. (**A**) Initial lifting of the ILM flap. (**B-C**) Progressive enlargement of the flap from the inferior to the superior margin. (**D-E**) Folding of the flap nasally over itself. (**F-G**) Final configuration of the flap, folded to cover half of the macular hole. (**H**) Final appearance after fluid–air exchange, performed with a soft tip in the nasal quadrant to stabilize the flap in the intended direction

This prospective, single-arm, interventional case series included patients undergoing primary surgical repair using the membrane loop giant ILM flap technique, as described below. Inclusion criteria consisted of a diagnosis of large (minimal linear diameter [MLD] > 400 μm or chronic (at least six months from initial diagnosis) IMH with no other ocular pathology aside from cataract. Patients with a history of intraocular surgery other than uncomplicated cataract extraction were excluded. All patients underwent a comprehensive preoperative ophthalmologic evaluation, including clinical exam, best-corrected visual acuity (BCVA) measured in Early Treatment of Diabetic Retinopathy Study (ETDRS) letters, and spectral-domain optical coherence tomography (SD-OCT) to assess the MLD, base diameter, and height of the macular hole. Surgery was performed using a standard 25-Gauge PPV. After core vitrectomy, the posterior hyaloid was visualized with micronized triamcinolone acetonide 40 mg/mL (Ophthaac 40^®^; Ophthalmos, São Paulo, Brazil), and the ILM was subsequently stained with Brilliant Blue 0.05% (Opht-Blue^®^; Ophthalmos, São Paulo, Brazil) to enhance contrast. An ILM flap measuring 2 to 3 mm in length was created exclusively with the membrane loop, without the use of forceps. The flap was initiated at the margin of the inferotemporal arcade and extended toward the edge of the macular hole (Fig. 1). After the fluid–gas exchange, with fluid aspiration performed on the nasal side of the optic disc and macula, the flap was displaced nasally following the flow created by this aspiration. As it moved, the flap folded over and completely covered the macular hole without being tucked underneath it. After meticulous removal of any residual fluid from the posterior pole, non-expansile C3F8 gas was injected into the vitreous cavity. All patients were instructed to maintain face-down positioning for four days postoperatively.

Postoperative evaluations were performed on days 1, 7, 14, 30, 60, and 180 using SD-OCT to assess macular hole closure and retinal morphology. The primary outcome was the anatomical closure rate on day 14. Secondary outcomes included change in BCVA, proportion of eyes with an improvement of ≥ 15 ETDRS letters, and the need for reoperation in six months. Statistical analyses were performed using Python, version 3.13 (Python Software Foundation; Wilmington, DE). Descriptive statistics are reported as medians and interquartile ranges (IQR). Differences in visual acuity between baseline and 6 months were assessed using the Wilcoxon signed-rank test. Results are presented as the median change with 95% confidence intervals (CI) obtained by bootstrapping.

The study was conducted in accordance with the tenets of the Declaration of Helsinki and was approved by the HCRP Institutional Review Board. Written informed consent was obtained from all participants prior to enrollment.

## Results

Eight eyes of eight patients (4 female, 4 male) with a median age of 69 years (range, 45–77 years) were enrolled. Six patients underwent surgery in the left eye, and two in the right eye. No intraoperative or postoperative complications were encountered. Baseline macular hole morphology was consistent with large, chronic disease: median MLD was 400 μm (IQR, 374–598), median base diameter was 915 μm (IQR, 645–1,210) and median height was 410 μm (IQR, 400–437). The corresponding median macular hole index was 0.46 (IQR, 0.45–0.49). Median symptom duration was 11 months (IQR, 10–21). Individual baseline characteristics are presented in Table 1.

**Table 1:** Cohort characteristics and summary of outcomes

	Gender	Age	Laterality	Symptoms Duration, mo.	MLD, µm	Height, µm	Base Diameter, µm	MHI	Baseline BCVA	Type of Flap	Closure 1st Surgery	Closure 2nd Surgery	6 Months PO BCVA
**Case 1**	F	66	Left	36	508	397	893	0.45	20/800	Temporal	Yes		20/80
**Case 2**	M	73	Left	18	688	375	1353	0.42	20/400	Temporal	No	Yes	20/200
**Case 3**	M	76	Right	12	849	433	1685	0.49	20/100	Inferior	No	Yes	20/80
**Case 4**	F	45	Left	10	258	541	639	0.61	20/200	Inferior	Yes		20/50
**Case 5**	M	77	Right	24	375	403	1066	0.45	20/200	Inferior	Yes		20/200
**Case 6**	F	66	Left	10	373	409	936	0.40	20/400	Temporal	No	Yes	20/63
**Case 7**	F	72	Left	10	420	410	650	0.46	20/400	Temporal	Yes		20/80
**Case 8**	M	65	Left	8	380	441	450	0.50	20/400	Temporal	Yes		20/32

Abbreviations: MLD – minimum linear diameter; MHI – macular hole index

### Anatomical outcomes

Six of eight macular holes (75%) were closed by day 14 after the primary intervention. Two cases did not close initially (Table 1, Cases 3 and 6), and one case recurred three months after initial closure (Table 1, Case 2). In all three eyes, postoperative day-1 OCT demonstrated flap displacement with incomplete foveal coverage, indicating loss of flap position rather than inadequate size or adherence. The recurrent case and the two that failed primary closure achieved closure after a second gas fill and flap repositioning with additional removal of the ILM nasal to the macular hole (see Supplemental Video), yielding a final closure rate of 100% at six months. No patient required reoperation beyond the second procedure, and no postoperative retinal detachments, flap dislocations, or endophthalmitis occurred.

### Functional outcomes

Visual acuity improved in seven eyes (87.5%) and remained stable in one. Median BCVA increased from 20 letters (IQR, 20–35) at baseline to 55 letters (IQR, 45–63) at six months, corresponding to a median improvement of 33 letters (95% CI, + 5 to + 48; *P* = 0.018). Six eyes (75%) achieved a ≥ 15-letter gain, and two eyes reached a Snellen equivalent of 20/63 (60 letters) or better.

## Discussion

In this prospective pilot series, the membrane loop-assisted giant ILM flap technique achieved high anatomical and functional success in the management of large or chronic IMH. Despite the challenging baseline characteristics of our cohort, with a median MLD of 400 μm and long symptom duration, anatomical closure was achieved in 75% of eyes, and all eyes attained final closure after reoperation, accompanied by meaningful visual improvement. These results support the feasibility and safety of using the membrane loop as a primary tool for ILM manipulation in macular hole surgery.

**Fig. 2:**
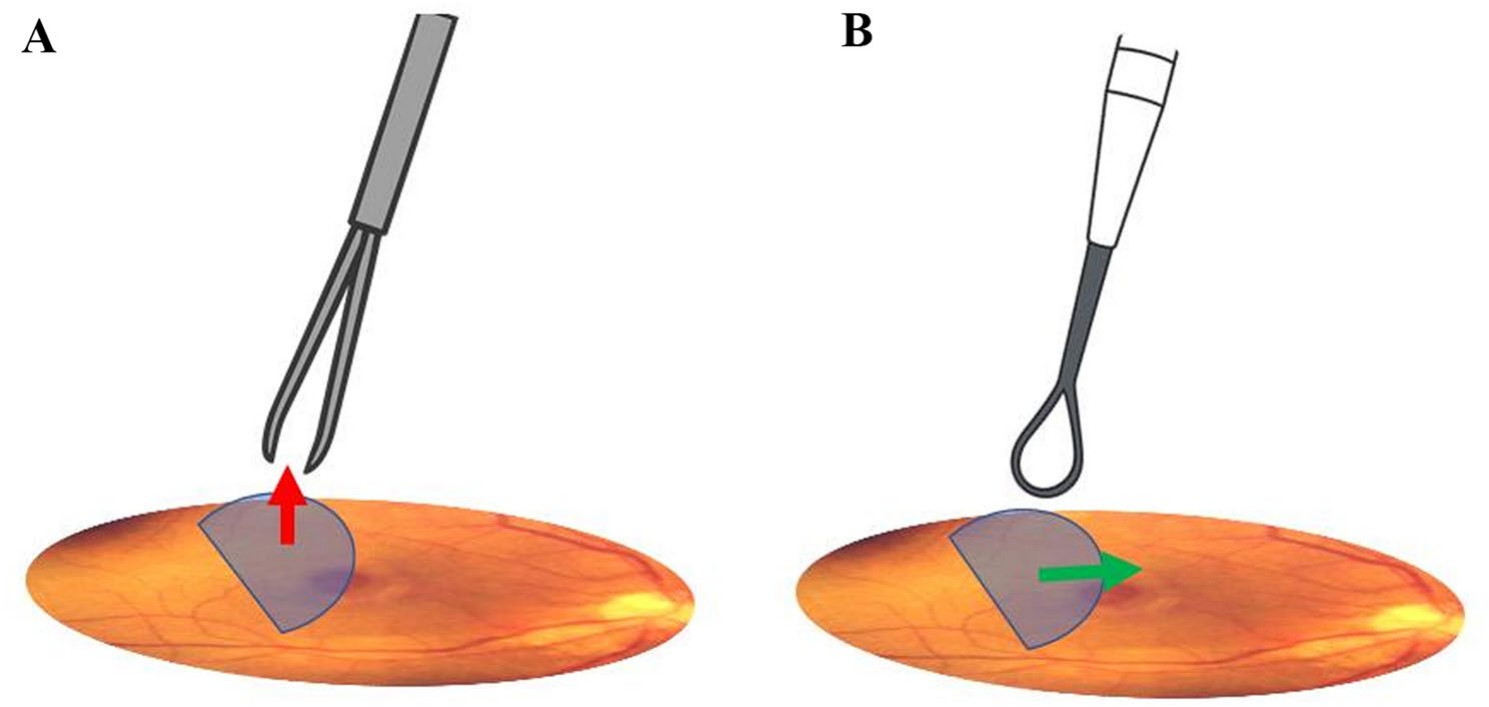
Schematic representation illustrating the anteroposterior force vector for flap construction using the pinch-and-peel technique (**A**). With the membrane loop technique (**B**), the force vector is tangential, which may reduce the risk of iatrogenic injuries

No intraoperative or postoperative complications were encountered. Unlike conventional forceps-based techniques, which rely on a “pinch and peel” maneuver utilizing anteroposterior force vectors that can cause focal trauma to the inner retina, the membrane loop allows for controlled, atraumatic dissection of the ILM using tangential forces (Fig. 2). Previous studies have associated forceps manipulation with dissociated optic nerve fiber layer (DONFL) changes and potential structural damage [11]. By reducing direct tractional stress on retinal tissue, the membrane loop may mitigate these risks, thereby supporting improved anatomical and functional outcomes [10]. In addition, based on our experience, the technique appears to offer a relatively short learning curve and reduced surgical time by allowing the same instrument to both lift and peel the ILM; however, these observations require confirmation in future comparative studies.

The primary closure rate of 75% in our series was comparable to the 51.9% reported by Kelly et al. in their pilot study on vitreous surgery for idiopathic macular holes [12]. However, it was lower than the outcomes described by Michalewska et al., who reported closure rates of 88% with conventional ILM peeling and 98% with the inverted ILM flap technique at the initial surgery [13]. Contemporary studies indicate that full-thickness macular holes smaller than 400 μm achieve closure in nearly all cases, whereas those larger than 400 μm close in approximately 80% [14, 15]. Even with more advanced approaches such as the inverted ILM flap, reported closure rates vary and are often influenced by surgical expertise [13, 16]. In our cohort, eyes requiring reoperation achieved complete closure after a single additional flap manipulation combined with gas tamponade, without necessitating more complex procedures such as amniotic membrane grafting or autologous retinal transplantation. These findings suggest that the giant ILM flap created with the membrane loop provides a stable scaffold, supporting delayed yet reliable closure.

The anatomical improvements were mirrored by functional gains. Six of eight eyes achieved a ≥ 15-letter improvement, and two eyes reached a Snellen equivalent of 20/63 or better. These results are particularly notable given that large, chronic macular holes are typically associated with limited visual prognosis [13, 16]. Preservation of retinal architecture and enhanced restoration of the outer retinal layers may help explain these results, suggesting that the membrane loop may have contributed to minimizing iatrogenic trauma. Although intraoperative OCT studies suggest that tangential membrane loop dissection may induce less focal retinal stress than the pinch-and-peel technique, our study was not designed to quantitatively compare rates of iatrogenic trauma, and future imaging-based comparative analyses are needed to address this question.

According to the Euler–Bernoulli beam theory, the deflection (*δ*) of a thin, elastic beam under bending is inversely proportional to its flexural rigidity (*E I*) and proportional to its length (*L*) [17]. Flexural rigidity depends on the material’s elastic modulus (*E*) and the area moment of inertia (*I*), while the length term scales strongly with geometry: $\:\delta\:\:\propto\:\:\frac{{L}^{3}}{E\:I}$. In the context of macular surgery, the ILM behaves as an ultra-thin elastic shee**t** that can be modeled locally as a beam. When creating an ILM flap, its stability over the macular hole depends on resistance to bending and unfolding.

Smaller ILM flaps have a shorter effective length (*L*) and, therefore, a lower bending stiffness, as the cubic term in the equation favors higher displacement. As a result, small flaps are more prone to folding back or dislodging from the hole, particularly under intraocular fluid currents and postoperative gas tamponade. Larger (giant) ILM flaps, in contrast, increase the effective length and surface area of contact with the retinal surface. Although the ILM itself is extremely thin, the larger arc of tissue increases mechanical anchoring and reduces the relative moment at the hinge, thus decreasing the tendency of the flap to curl or unfold. In practical terms, the larger flap has greater geometric stability, distributing mechanical forces more evenly and resisting displacement.

Furthermore, larger flaps act not only as a mechanical cover but also as a biological scaffold, facilitating gliosis and migration of Müller cells [13, 18]. This dual effect of mechanical stability from elasticity principles and biological support provides the rationale for employing giant ILM flaps created with the membrane loop, which allows atraumatic dissection of wide flaps without the microtrauma associated with forceps.

This preliminary study has several limitations. The small sample size and the absence of a comparator group limit the generalizability of our findings and preclude definitive conclusions regarding the superiority of the membrane loop relative to conventional forceps-based techniques. As an exploratory pilot investigation, the study was primarily designed to assess the feasibility, safety, and short-term anatomical and functional outcomes of membrane loop–assisted giant ILM flap creation; therefore, no formal sample size or power calculation was performed. These preliminary findings should be interpreted with caution and validated in larger, randomized controlled trials.

## Conclusions

Taken together, our results suggest that membrane loop–assisted giant ILM flap creation may be a feasible, reproducible, and safe strategy for the treatment of large and chronic IMH. While limited by the small sample size and non-comparative design, the 75% primary and 100% final closure rates and consistent visual improvement observed in this series warrant further validation in larger, randomized studies. If confirmed, the membrane loop may represent a valuable advancement in the surgical armamentarium for macular hole repair.

## Supplementary Information

Below is the link to the electronic supplementary material.

Supplementary Material 1

## Data Availability

Anonymized data used and/or analyzed during the current study are available from the corresponding author on reasonable request.

## Abbreviations

IMH: Idiopathic macular hole
PPV: Pars plana vitrectomy
ILM: Internal limiting membrane
MLD: Minimal linear diameter
BCVA: Best–corrected visual acuity
ETDRS: Early Treatment of Diabetic Retinopathy Study
SD: OCT–Spectral–domain optical coherence tomography
IQR: Interquartile range
CI: Confidence interval
DONFL: Dissociated optic nerve fiber layer
